# Interleukin-17 in Chronic Inflammatory Neurological Diseases

**DOI:** 10.3389/fimmu.2020.00947

**Published:** 2020-06-03

**Authors:** Jelena Milovanovic, Aleksandar Arsenijevic, Bojana Stojanovic, Tatjana Kanjevac, Dragana Arsenijevic, Gordana Radosavljevic, Marija Milovanovic, Nebojsa Arsenijevic

**Affiliations:** ^1^Faculty of Medical Sciences, Center for Molecular Medicine and Stem Cell Research, University of Kragujevac, Kragujevac, Serbia; ^2^Department of Histology and Embriology, Faculty of Medical Sciences, University of Kragujevac, Kragujevac, Serbia; ^3^Department of Pathophysiology, Faculty of Medical Sciences, University of Kragujevac, Kragujevac, Serbia; ^4^Department of Dentistry, Faculty of Medical Sciences, University of Kragujevac, Kragujevac, Serbia; ^5^Department of Pharmacy, Faculty of Medical Sciences, University of Kragujevac, Kragujevac, Serbia

**Keywords:** IL-17, Th17, EAE, Alzheimer's disease, ischemic brain injury

## Abstract

A critical role for IL-17, a cytokine produced by T helper 17 (Th17) cells, has been indicated in the pathogenesis of chronic inflammatory and autoimmune diseases. A positive effect of blockade of IL-17 secreted by autoreactive T cells has been shown in various inflammatory diseases. Several cytokines, whose production is affected by environmental factors, control Th17 differentiation and its maintenance in tissues during chronic inflammation. The roles of IL-17 in the pathogenesis of chronic neuroinflammatory conditions, multiple sclerosis (MS), experimental autoimmune encephalomyelitis (EAE), Alzheimer's disease, and ischemic brain injury are reviewed here. The role of environmental stimuli in Th17 differentiation is also summarized, highlighting the role of viral infection in the regulation of pathogenic T helper cells in EAE.

## Introduction

Interleukin-17 (IL-17) is the first-described and founder member of the IL-17 family of inflammatory cytokines, which contains six members: IL-17A, IL-17B, IL-17C, IL-17D, IL-17E, and IL-17F. The gene that encodes IL-17A was discovered in 1993 as an RNA transcript homologous to a *Herpesvirus Saimiri* gene, and the protein, initially called CTLA-8, was cloned (1). However, IL-17 attracted widespread attention in 2005, when two independent groups simultaneously characterized a new population of T helper (Th) CD4+ cells that produced IL-17A, named Th17 (2, 3). T helper CD4+ cells were first marked as the principal source of IL-17, but it was later shown that CD8+ cells also produce this cytokine, and these cells are termed Tc17. Also, several types of innate immune cells such as γδ T, natural killer T (NKT), TCRβ+ natural Th17, and Type 3 innate lymphoid cells (ILC3) produce IL-17 (4). All of these IL-17-producing cells are termed “Type 17” cells.

The proinflammatory activities of IL-17 are key in anti-microbial protection of the host, but uncontrolled IL-17 activity is associated with different immunopathological conditions, autoimmune diseases, and cancer progression (5). A critical role for IL-17R signaling in protection against bacterial and fungal infections, particularly by Candida albicans and Klebsiella pneumoniae, has been described in various studies in mice (6). In humans, mutations in IL-17 signaling genes (ACT1, IL17RA, IL17RC) are associated with chronic mucocutaneous candidiasis (5, 7, 8). The same condition also develops in individuals with AIRE deficiency, a condition accompanied by the production of anti-IL-17 antibodies (9).

Anti-IL-17A antibodies have shown therapeutic effect in various inflammatory diseases. Several anti-IL-17 antibodies have been approved for the treatment of plaque psoriasis (10, 11). Positive effects of IL-17 blockade have been shown in clinical trials of ankylosing spondylitis and psoriatic arthritis (12). Anti-IL17R antibody treatment of Crohn's disease has been shown to worsen the disease (13, 14), whereas targeting cytokines that control the differentiation of Th17 cells and therefore IL-17 secretion with anti-p40 subunit antibodies (Ustekinumab, Briakinumab) and anti-IL-6 receptor antibody (Tocilizumab) showed efficacy (15–17). These findings indicate that IL-17, by maintaining the integrity of the intestinal barrier, plays a dominantly protective role that overcomes its potential for tissue destruction in inflammatory bowel disease (18). Clinical use of antibodies that target IL-17 signaling gave insights into functions of IL-17 in humans.

## IL-17R Signaling

The family of IL-17 receptors contains five different receptors (IL-17RA, IL-17RB, IL-17RC, IL-17RD, and IL-17RE) with common a cytoplasmic motif known as the SEFIR domain (19). IL-17 exists either as a homodimer or as a heterodimer, and both forms of the cytokine induce signals through dimeric IL-17RA and IL-17RC receptor complex (5). Binding of IL-17 to its receptor induces activation of several independent signaling pathways mediated by a cytosolic adaptor protein, Act1, and different TRAF proteins (5, 19, 20). IL-17 signaling mediated through TRAF6 and TRAF4 results in the transcription of inflammatory genes. Activation of TRAF6 by binding of IL-17 to its receptor leads to triggering of NF-κB, C/EBPβ, C/EBPδ, and MAPK pathways, while TRAF4 activation in complex with MEKK3 and MEK5 activates ERK5 (21). On the other hand, the mRNA stability of genes controlled by IL-17 is controlled IL-17-activated TRAF2 and TRAF5 (22).

Expression of IL-17R is ubiquitous, but the main targets of IL-17 are non-hematopoietic cells (23). IL-17 signaling induces the production of proinflammatory cytokines (IL-1, IL-6, G-CSF, GM-CSF, and TNF) and chemokines (CXCL1, CXCL2, CXCL5, CCL2, CCL7, CCL20, and IL-8), matrix metalloproteinases (MMP1, MMP3, MMP9, and MMP13), and anti-microbial peptides (β-defensins, S-100 proteins) (24, 25). The biological activities of IL-17 are often the result of synergistic or cooperative effects of IL-17 and other inflammatory cytokines (26). There are several mechanisms of negative regulation of IL-17 signal transduction. The negative regulators of IL-17 signaling are different ubiquitinases, deubiquitinases, kinases, endoribonuclease, and micro RNAs (21).

However, there is tissue-specific IL-17-dependent gene induction (27). In gut epithelium, IL-17 regulates the expression of several molecules that contribute to the preservation of continuous intestinal epithelium. In renal epithelial cells, IL-17 induces the expression of kallikrein 1 (28), while in salivary epithelium, it induces the expression of histatins (29), molecules that are involved in protection against *C. albicans*. IL-17-mediated osteolysis, which is detected in periodontitis and mouse models of arthritis or periodontal disease, is probably mediated by a receptor activator of NF-κB ligand (RANKL, TNFSF11, or osteoprotegerin ligand, OPGL) whose espression is induced by IL-17 (30, 31).

## Differentiation of TH17 Cells

Th17 cells are classified as an inflammatory subset of T helper cells that perform key roles at mucosal surfaces where they mediate protection from bacteria and fungi and also contribute to the regulation of the mutualistic microorganisms that constitute the microbiota (32, 33). However, Th17 cells are also one of the major factors in the pathogenesis of several autoimmune diseases, including autoimmune disease of the central nervous system, Multiple sclerosis (MS) (2, 3, 34–39). The process of differentiation of naive CD4+ cells into Th17 cells is very similar to that of Th1 differentiation, but transcriptional factors that mediate this are distinct and it require stimulation with the cytokines IL-1β, IL-6, IL-21, and TGFβ, which are produced by professional antigen-presenting cells (APCs) (32, 40–47). Cytokines produced by APCs stimulate the JAK-STAT3 axis and upregulate the expression of transcription factors RORγt and RORα, identified as markers of the Th17 lineage (48–52). The differentiation of Th17 cells is reduced in the states of IL-6, IL-21, TGFβ, or RORγt deficiency, which leads to reduced production of Th17 cytokines and impaired defense against extracellular bacteria and fungi but also attenuation of autoimmunity (41, 48, 53). However, an alternative mode for the differentiation of pathogenic Th17 cells in the absence of TGFβ signaling has been described *in vivo* in Experimental Autoimmune Encephalomyelitis (EAE) (54). Cytokines that induce Th1 and Th2 differentiation are described as the main inhibitors of Th17 differentiation. IL-2 is a key repressor of Th17 differentiation, as it activates transcription factor STAT5 and thus inhibits IL-17 production (55), while inhibition of IL-2 expression in T lymphocytes stimulates Th17 cell development (56, 57).

In animal models of autoimmune diseases, proinflammatory cytokines IL-1β and IL-23 have been shown to be enhancers and stabilizers of partially or completely differentiated effector Th17 cells, which dominantly express corresponding receptors for these cytokines, IL-1R1 and IL-23R (44, 58–61). In line with this observation, transfer of Th17 cells *in vitro* obtained by exposure to IL-6 and TGFβ does not induce EAE in mouse, while Th17 cells obtained by stimulation of naive cells with IL-1β, IL-6, and IL-23 achieve the pathogenic potential and are able to elicit EAE (55). In fact, it was later shown that IL-6 and TGFβ in Th17 cells induce production of anti-inflammatory cytokine IL-10, while IL-23 has a critical role in the induction of the endogenous cytokine TGFβ3. Suppression of IL-10 production in Th17 cells during their differentiation results in high expressions of T-bet, IL-23R, and GM-CSF, markers of Th17 cells with pathogenic potential (62, 63). Furthermore, IL-1 and IL-23 stimulation through JunB and SOCS family members (64, 65) affects the effector profile of Th17 cells and induces the development of highly pathogenic double-positive IL-17+ IFNγ+ and IL-17+ GM-CSF+ T cells (66–68). These pathogenic double-positive cells originate from Th17 cells. However, IL-23 is not required for the differentiation and maintenance of nonpathogenic Th17 cells in the gut and functional plasticity toward T follicular helper cells (66, 68). The novel genes Gpr65, Toso, and Plzp, identified by the single-cell RNA-sequencing analysis of *ex vivo* Th17 cells, are found to promote Th17 pathogenicity and to cause EAE and chronic inflammation in the CNS of mice, while CD5 antigen-like (CD5L) attenuates Th17 cell-mediated disease (69, 70).

Before activation, T cells do not express receptors for cytokines IL-1 and IL-23 (58, 71). During the initial phase of Th17 differentiation, IL-6 induces binding of RORγt to the Il1r1 locus and binding of STAT3 to the Il23r locus, leading to the expression of these genes (54). Phosphorylation of STAT3 increases the expression of the conserved miR-183/96/182 cluster, which in turn reduces the expression of Foxo1, a transcription factor that negatively regulates the expressions of IL-1R1 and IL-23R (72). The Major Transcriptional Effector of Notch Signaling, RBPJ, promotes IL-23R expression and induces pathogenicity of Th17 cells (73). The differentiation of Th17 is stabilized by positive feed-forward loop stimulation with IL-1β and IL-23, accompanied by upregulation of IL-1R and IL-23R (50, 74). The hallmark of effector Th17 cells is IL-23R expression, and its signaling promotes the expression of transcriptional factor Blimp-1, which induces the expression of several genes *in vivo*, leading to the enhanced pathogenicity of Th17 cells (75).

The differentiation of human Th17 cells *in vitro*, similar to mouse Th17 cells, requires IL-1, IL-6, IL-23, and TGFβ. Initially, a few studies demonstrated that TGFβ was not required, while stimulation with IL-1β, IL-6, and IL-23 was sufficient for induction of human Th17 cell differentiation (76, 77). Later studies showed that TGFβ, IL-23, and IL-1β (or IL-6) were the key factors needed for differentiation of human Th17 cells under serum-free conditions, since serum could be the source of TGFβ or aryl hydrocarbon receptor (AhR) ligands (78, 79). In line with findings regarding Th17 differentiation in mouse, IL-23 is also the key player in the differentiation of human Th17 cells; moreover, human CD4+ T cells express IL-23R before activation and immediately respond to IL-23, while IL-23 signals in accordance with stimulation with IL-1β further upregulate IL-23R expression (78, 80). Described dominant-negative mutations of STAT3 gene, which can be manifested by hyper-immunoglobulin E syndrome, inadequate Th17 cell differentiation, and reduced production of IL-17, supports the roles of STAT3 in the IL-6- and IL-23-mediated process of Th17 differentiation in humans (81, 82).

## Environmental Factors That Affect the Pathogenic Potential of Th17 Cells

Different environmental factors modulate the reactions of the immune system and strongly accelerate the pathogenic potential of Th17 cells. T helper cells under Th17 culture condition increase expression of the aryl hydrocarbon receptor (AhR), a ligand-dependent transcription factor that senses environmental toxins and endogenous molecules such as metabolites of tryptophan, and the stimulation of this molecule induces the release of IL-17 and IL-22 by effector Th17 cells (83, 84). The transcription factor, hypoxia-inducible factor 1 (HIF-1), a key metabolic sensor, directly regulates expression of RORγt and IL-17 at the transcriptional level and promotes Th17 differentiation (85, 86). Signaling *via* kinase complex mTORC1 coordinates metabolic and transcriptional programs that regulate the development of pathogenic Th17 cells (87). Disrupted mTORC1 signaling in Th17 cells leads to upregulated expression of TCF-1 (transcription factor T-cell factor 1) and development of stemness-like features, while transdifferentiation in the Th1 is arrested. Mice with blocked mTORC1 activity are protected from EAE, while their Th17 cells do not express T-bet and IFN-γ (87).

A high-salt diet can enhance the differentiation of Th17 cells and thus contribute to the development of EAE (88, 89). Exposure to a high-salt diet induces the expression of serum glucocorticoid kinase 1 (SGK1), which promotes the expression of IL-23R and thus stabilizes pathogenic Th17 cells and enhances the production of GM-CSF (88, 89). Since mice with T-cell-specific deletion of *Sgk1* develop attenuated EAE, without exacerbation after exposure to a high-salt diet, it appears that a high-salt diet modulates EAE severity by its direct effect on T-cell differentiation (88).

## Th17 and IL-17 in Multiple Sclerosis and EAE

Multiple sclerosis is a chronic inflammatory disease of the central nervous system (CNS) that is characterized by damage to myelinated axons in the CNS, leading to the loss of myelin sheath. Inflammatory processes that cause myelin damage lead to the destruction of oligodendrocytes and axons, with subsequent axonal loss, and transient or permanent loss of neurologic functions, resulting in various types of disabilities of different severity (90). An overall reduction in CNS volume is very often seen in MS. Localized inflammatory foci can be found in the white matter in almost all areas of CNS, with a considerable number of plaques in the gray matter and anywhere in the CNS parenchyma, including the optic nerves, brainstem, periventricular white matter, and cervical spinal cord (91–93). The course of MS and clinical symptoms are highly variable and unpredictable, varying from a relatively benign illness with minimal impairment to a rapidly evolving and life-threatening disease that requires serious medical treatment (94).

The precise etiology of MS is unknown, but it is considered that both genetic and environmental factors play significant roles in its development (95). The pathogenesis of MS also remains elusive, but it is believed that MS is an autoimmune disease mediated by auto-reactive CD4+ T cells specific for myelin antigens. Autoreactive T cells initiate and perpetuate an inflammatory cascade, resulting in demyelination and axonal loss (96). The huge heterogeneity of disease course in patients with MS and in the histopathological features seen in the CNS indicates that multiple immunopathological pathways contribute to the disease development. Evidence from clinical studies suggests that inflammatory mediators, such as cytokines, play an essential role in the pathogenesis of MS (91, 97).

The pathogenesis of MS has been mostly described by analogy to EAE, an animal model of MS (98–100). In typical EAE induced by immunization with autoantigen, myelin-specific CD4^+^ T cells are activated in the lymph organs in the periphery, develop encephalitogenic potential, and infiltrate the CNS, where they recognize specific autoantigens presented by local antigen-presenting cells (APCs) and reactivate. The inflammatory process in MS is initiated by binding of pathogen-associated molecular patterns (PAMPs) from pathogens or commensal bacteria and damage-associated molecular patterns (DAMPs) from dead or dying cells to pathogen recognition receptors (PRRs), leading to activation of innate immune cells and production of IL-1, IL-6, IL-12, IL-18, and IL-23, cytokines that promote the differentiation and expansion of encephalitogenic Th1 and Th17 cells (101, 102). Myelin-specific CD4+ T cells that enter the CNS are reactivated and expanded by the IL-1β and IL-23 produced by resident microglia and infiltrating inflammatory monocytes. Encephalityogenic Th1 and Th17 cells in the CNS produce inflammatory cytokines that activate glial cells to produce inflammatory mediators, matrix metalloproteinases, chemokines, and free radicals, which induce myelin damage, leading to manifestations of neurologic deficits (65, 66, 101, 103). One of the main differences between MS and animal models (EAE) is the localization of demyelination. In EAE demyelination, is mainly located in the spinal cord, whereas in MS, this process mainly affects the cerebral and cerebellar cortex (104). The dominant population of T cells in active MS lesions are CD8+ T cells, but in EAE, the primary encephalitogenic T cells and dominant population in CNS infiltrates are CD4+ T cells, with less evidence for the role of CD8+ T cells (105). Neurodegeneration is more typical for MS, while in EAE models, the dominant finding is neuroinflammation (106). In the later stages of MS, neurodegeneration appears to be independent of the inflammatory process, which cannot be found in the acute inflammatory EAE model (107). However, axonal and neuronal loss and demyelination with remyelination can be observed in EAE in Biozzi antibody high (ABH) mice (108). Despite the limitations of the EAE models, the main findings regarding MS pathogenesis have come from EAE studies, as has the design, development, and validation of many therapeutics used for the treatment of MS (109).

Cytokines play roles in the pathogenesis of MS and EAE and in the processes of inducing oligodendrocyte cell death, neuronal dysfunction, and axonal degeneration (110). Th17 cells are considered to be one of the key effectors of autoimmune inflammatory diseases, including MS and experimental disease EAE (2, 111–113). Increased expression of IL-17- and Th17-associated transcripts (Il6, Il17a) has been demonstrated in MS plaques collected at autopsy (114). Further, IL-17 was marked as the highest-ranking gene expressed in the CNS of MS patients at autopsy (114); this was before the discovery of Th17 cells. Also, another report indicated that MS is a primarily IL-17-mediated autoimmune disease (78). Later, the results of various studies showed that a single nucleotide polymorphism (SNP) in IL-23R gene is linked to several human autoimmune diseases, indicating that IL-23 signaling is an essential event in the development of pathogenic Th17 cells. It is known that IL-17 can stimulate the production of other proinflammatory cytokines and chemokines and thus evince a powerful proinflammatory effect (115). The concentration of IL-17 is significantly higher in the serum of MS patients with relapses and remissions than in normal, healthy subjects (116) and is in correlation with disease activity, as demonstrated by magnetic resonance imaging (117). Consistently with the increased concentration of IL-17 in liquor and peripheral blood of MS patients, the proportion of Th17 cells is also increased, especially during relapses, while there is no change in Th1 cells (118, 119). Th17 cells are able to cross the blood–brain barrier, and their presence in MS lesions is associated with enhanced neuroinflammation (120). It has been shown that IFN-γ-producing Th17 cells cross the blood–brain barrier and accumulate in the CNS during the active phase of MS (121). Besides CD4+ T cells, there is evidence that IL-17-producing CD8 T cells contribute to CNS tissue damage in EAE and are also present in the liquor of patients with MS (122, 123). Importantly, it has been documented that the cells that enter the CNS in the first wave of CNS infiltration are Th17 cells (124), followed by infiltration with other immune cells that further promote and sustain tissue inflammation. Also, the presence of IL-17- and IL-22-producing Th cells has been reported in the early stages of MS (125).

Beneficial effects of treatment with rituximab, blocking anti-CD20 antibody, in EAE are associated with decreased production of several cytokines, including IL-17 (126). Neutralization of IL-17 can significantly attenuate the progress of EAE by attenuating the induction of pathogenic cytokines (58). Also, EAE severity was ameliorated in IL-17-deficient animals (123, 127, 128), while the disease was mild, with delayed onset, in RORγt-deficient mice (48).

One study indicates that the beneficial effect of vitamin D supplementation in MS patients is mediated by alleviating the percentage of pathogenic T-cell subsets that produce IL-17 (129). It has also recently been shown that amelioration of MS by dimethyl fumarate is associated with suppression of IL-17+ CD8+ Tc17 cells (130). The beneficial effect of statins in some forms of MS could be due to their effect on Th17 cells (131). Phase IIa study has been conducted in order to investigate possible beneficial effects of Secukinumab, an IL-17A-neutralizing monoclonal antibody. No adverse effects of Secukinumab were detected, while the results of this study indicate that blocking IL-17A with an antibody may reduce MRI lesion activity in MS (132).

There are studies that demonstrate the importance of Th17 cells in EAE and MS, but there is also evidence that indicates that Th1 cells are the main mediators of neuropathology in the EAE model (113, 133). Several reports indicated that IFN-γ-deficient and IFN-γR-deficient mice, as well as anti-IFN-γ-treated mice, develop EAE (134, 135). Also, there are reports showing a protective role for IFN-γ in EAE, mediated by the supression of pathogenic Th17 cells (3). The presence of T cells that coexpress IL-17 and IFN-γ under inflammatory situations has been reported (136, 137). These Th1/Th17 cells were noticed in the CNS of mice with EAE (138). Data obtained from the mouse studies indicate that Th17 cells lacking Il17a generated *in vitro* are able to induce EAE upon adoptive transfer, similar to wild-type Th17 cells (127, 139). Finally, it seems that there is significant plasticity of Th17 cells, with evidence that lymphocytes obtained from the blood of MS patients have an increased potential to switch from IL-17-secreting Th17 cells to IFN-γ-secreting Th1, also called ex-Th17 cells (121).

Several studies have demonstrated that IL-23, a cytokine essential for the differentiation and expansion of Th17 cells, promotes EAE more robustly than IL-12, a cytokine that stimulates the development of INF-γ-producing Th1 cells (140). IL-23 is a covalent heterodimer of p40 (IL-12) and p19 (IL-23) subunits (70). IL-12 and IL-23 share the p40 subunit. The same cell types, mainly dendritic cells, produce both of these two cytokines, but their relative ratio depends on the nature of stimuli that activate dendritic cells (141). IL-12Rβ2-deficient mice with excluded IL-23 signaling are more susceptible to EAE, develop disease earlier, and have more severe disease, with greater demyelination and CNS inflammation, compared to WT mice (142). This result was contrary to findings in IL-12Rβ1-deficient mice (excluded IL-12 signaling) (143). Also, it has been shown that IL-23, not IL-12, plays the key role in the development of CNS autoimmune inflammation, affecting the subset of memory Th1 cells (144). It has also been shown that IL-23 induces differentiation of highly encephalitogenic Th cells that produce IL-17A (145).

In attempts to clearly define the roles of Th1 and Th17 subpopulations in MS pathogenesis, it was shown that the transfer of Th17 cells induces more severe EAE compared to Th1 cells (58). Another study showed that autoantigen-specific Th1 and Th17 cells were able to induce disease with similar severity but with different pathological findings (133). Th1-mediated neuroinflammation was characterized by macrophage infiltration, while, in Th17-mediated disease, neutrophil predominated in CNS infiltrates (133). Also, it was found that Th17 cells induce mainly brain damage, in contrast to Th1 cells, which dominantly induce spinal cord inflammation (146).

IL-17 mediates EAE development by the stimulation of IL-17R expressed on endothelial cells, astrocytes, microglia, and resident neuroectodermal cells (147). Mouse astrocytes express receptor for IL-17 (148) when stimulated with recombinant IL-17A *in vitro*, but also, *in vivo* in the EAE model, they produce various cytokines and chemokines, IL-6, TNFα, CCL2, CCL3, CCL20, CXCL1, CXCL2, CXCL9, CXCL10, and CXCL11 (IP-9) (149–151), that promote the influx of immune cells into the CNS and mediate neuroinflammation. Similarly, human astrocytes cultured with IL-17 *in vitro* produce IL-6, a cytokine that perpetuates the differentiation of CD4 naive cells into Th17 cells (152). The role of IL-17-mediated activation of astrocytes in EAE pathogenesis was confirmed by attenuation of EAE in animals with blocked IL-17 signaling in astrocytes (152). IL-17 also contributes to EAE development by affecting the activity of NG2+ oligodendrocyte precursor cells (OPCs) (153). Further, *in vitro* treatment of these cells with IL-17 strongly inhibits the maturation of oligodendrocytes and reduces their survival (154). Another study also indicates that IL-17 mediates apoptosis and inhibits differentiation of oligodendrocytes *in vitro* (155). IL-17 stimulates the maturation of primary OPCs and their participation in inflammatory processes (156). Microglial cells stimulated *in vitro* with IL-17 produce inflammatory mediators IL-6 and CXCL2, while only LPS pre-stimulated microglia exert enhanced cytotoxic effects (157). Further, microglial cells co-cultured with Th1/Th17 cells, but not Th1-only cells, produce high amounts of IL-1β, IL-6, and TNF-α, which promote further Th17 differentiation, neuroinflammation, and damage (158). IL-17 disrupts blood–brain barrier (BBB) tight junctions *in vitro* and *in vivo* in MS and promotes CNS inflammation (120). In an EAE model, it has been shown that IL-17 disrupts BBB by the induction of oxidative stress in endothelial cells accompanied by down-modulation of the tight junction molecule occludin (159). IL-17A levels are elevated in the CSF of relapsing-remitting MS patients, and this level correlates with the level of BBB dysfunction. Also, the treatment of BBB cell line hCMEC/D3 with a combination of IL-17A and IL-6 reduces the expression of tight junction-associated genes and disrupts monolayer integrity (160). Indirect evidence supports the role of IL-17 in direct neuronal damage. Different neuronal populations express IL-17 receptor (161). Direct contact, resembling immune synapses, of MOG-specific Th17 cells and neurons in demyelinating lesions associated with axonal damage has been shown by confocal, electron, and intravital microscopy, indicating the central role of Th17 cells in neuronal dysfunction (162).

IL-22, a cytokine whose production specifically induces IL-23, contributes to the pathogenicity of Th17 cells (163). It has been reported that IL-22 contributes to MS severity (120) as well as dysregulated expression of IL-22 and its antagonist, IL-22BP (164). Single nucleotide polymorphism in the IL-22R A2 gene is associated with MS risk (91, 165). IL-22 can contribute to MS pathogenesis by enhancing the expression of Fas in oligodendrocytes, resulting in oligodendrocytic apoptosis, and decreasing the expression of FOXP3 in T cells (166). Production of IL-22 is increased during the peak phase of EAE and is decreased during remission (167). However, beside involvement in many neurological inflammations, IL-22 may also be protective (168).

Although Th17 cells and their hallmark cytokines IL-17, IL-22, and IL-23 have been marked as the crucial players in the pathogenesis of MS and EAE, however, mice lacking IL-17 and IL-22 develop EAE (62, 169).

Findings indicating that GM-CSF has the key role in the encephalitogenic potential of Th17 cells in mice (74, 170, 171), specifically, increased levels of GM-CSF in the cerebrospinal fluid and serum of active MS patients with the relapsing-remitting type of the disease and increased secretion of GM-CSF from T cells isolated from the peripheral blood and brain lesion of MS, suggest that GM-CSF also plays an important role in MS development (172, 173). Unlike other cytokines, GM-CSF plays a non-redundant role in EAE development, and its secretion alone is able to provide development of autoaggressive and pathogenic MOG-specific T cells (170). GM-CSF-deficient Th cells are not able to induce EAE, indicating that the encephalitogenic potential of both Th1 and Th17 cells depends on their GM-CSF production (74).

In our previous studies, we have shown that overcoming resistance to induction of EAE with MOG_35−55_ peptide of BALB/c mice by infection with murine cytomegalovirus (MCMV) (174) or by deletion of ST2 gene (169) is associated with increased production of IL-17 in T cells.

Disease developed by MCMV-infected BALB/c mice is accompanied with an increase in IL-17-positive CD4+ and CD8+ cells in the central nervous system. Brain infiltrates in MCMV-infected BALB/c mice were more significant that in C57BL/6 mice, with a similar number of CD4+ and CD8+ cells, contrary to the dominantly CD4+ cells in C57BL/6 mice, which develop “typical” EAE (105). The encephalitogenic potential of CD4+ T cells in the CNS infiltrates of BALB/c mice is further documented by the detection of CCR6, the key molecule that mediates the initial infiltration of the CNS by Th17 cells (174, 175). Almost equal participation of IFN-γ- and T-bet (Th1)- and IL-17- and RORγt (Th17)-expressing cells was found in the CNS of MCMV-infected MOG_35−55_ immunized BALB/c mice, in contrast to almost exclusive CNS infiltration with Th1 cells in C57BL/6 mice infected with γHV-68 before EAE induction (174, 176). Further, CNS infiltrates of BALB/c mice infected with MCMV before MOG_35−55_ immunization contained CD8+ cells that express T1 and T17 transcriptional factors and corresponding cytokines, TNF-α and IFN-γ (Tc1) and IL-17 (Tc17 cells) (174).

Since cerebrospinal fluid of early-stage MS patients contains a greater number of Tc17 cells in comparison with peripheral blood, these cells are considered to be required for the accumulation of Th17 cells in the CNS in MS (177). No inflammatory T1 and T17 cells were found in the CNS of BALB/c mice immunized with MOG_35−55_ (174), while in the CNS of unimmunized BALB/c mice infected with MCMV neonatally, Tc1 cells (IFN-γ and T-bet+) dominated (178). CD8+ T cells isolated from CNS of MCMV-infected and MOG_35−55_-immunized mice produced inflammatory cytokines in response to *in vitro* MOG_35−55_ peptide stimulation but were not specific for viral epitopes pp89 and m164 (174). These findings indicate that the newly developing autoimmune process in MOG_35−55_-immunized BALB/c mice previously infected with MCMV attracts a new population of IL-17-producing CD8+ cells that participate in the development of autoimmunity (177). These findings are in line with previous reports that the expansion of myelin-specific CD8+ T cells follows CD4+ T cell-mediated initiation of the autoimmune process in CNS, thus contributing to tissue damage (179). The significant presence of IL-17-, CCR6-, and RORγt-positive CD4+ and CD8+ cells in the CNS of MOG_35−55−_immunized BALB/c mice with non-productive MCMV infection in contrast to uninfected BALB/c mice immunized with MOG_35−55_, with negligible number of these cells in the CNS, indicates that MCMV infection probably modulates the activation and differentiation of antigen-presenting cells in the periphery, changing their signature cytokines, and thus, after additional stimulus, enables the development of Th17/Tc17 cells with encephalitogenic potential (174).

Our results indicate that MCMV infection of BALB/c mice significantly affects dendritic cells in peripheral lymph nodes, thus enabling differentiation of encephalitogenic cells (174). In line with the well-known capacity of MCMV to encode an analog of chemokine CCL2 (180) that induces monocyte recruitment and viral dissemination (181), we found a higher percentage of CCR2+ dendritic cells in the peripheral lymph nodes of MCMV-infected mice (174). In contrast with a previous report that MCMV attracts monocytes that acquire immunosuppressive characteristics (182), we found higher percentages of dendritic cell-expressing markers of activation, CD86 and CD40, and Th1-promoting cytokine IL-12, indicating that MCMV infection of BALB/c mice increases the proportion of inflammatory dendritic cells in peripheral lymph nodes and thus enables the development of encephalitogenic T cells (174).

## IL-17 in Alzheimer's Disease

Alzheimer's Disease (AD) is the most common neurodegenerative disorder causing cognitive impairment in the elderly (183, 184). The histopathological hallmarks of AD are amyloid plaques in the brain, mainly consisting of fibrillary forms of amyloid β peptide-40 (Aβ-40) and amyloid β peptide-42 (Aβ-42) (185). The fibrillary forms of amyloid β found in the amyloid plaques are obtained by a sequential cleavage from amyloid precursor proteins (186, 187). Highly insoluble Aβ peptides generated in the CNS play a crucial role in the pathogenesis of AD; they activate the complement pathway (168) and stimulate microglia to produce the proinflammatory cytokines and chemokines and thus induce accumulation of inflammatory cells into the CNS (188, 189). This proinflammatory process mediated by microglia leads to neurodegeneration (188, 190), although microglia play a protective role also, due to the clearing of Aβ aggregates by phagocytosis (191). Aβ peptides also increase the production of reactive nitrogen and oxygen species by microglial cells, leading to oxidative stress development, stimulation of Th17 cells, and IL-17 production (192, 193). It appears that the main roles of IL-17 in AD pathogenesis are the attraction of neutrophils and the stimulation of their function. It has been shown that Aβ aggregates mediate the chemotaxis and the recruitment of neutrophils in the CNS of mice overexpressing human mutant amyloid precursor protein (APP), which produce IL-17 and thus amplify neutrophil entry in the CNS (192), although mesenteric lymph nodes of these mice have lower production of IL-17 as a consequence of reduced differentiation of Th17 cells (194). Since neutrophils are the main targets of IL-17 in the CNS but are also very important sources of this cytokine, these cells, by promoting inflammation and CNS tissue damage, could have an important role in the development of AD pathology. Results from *in vitro* experiments indicate that IL-17 might promote autophagy in neurons and thus induce neurodegeneration (195).

There have been more reports about the role of innate immunity in AD than about adaptive immunity, but increased activation of T and B lymphocytes was recently demonstrated in a triple transgenic mouse model that replicated Aβ and tau neuropathology (196). Moreover, it has been shown that these cells produced high levels of IL-2, TNF-α, IL-17, and GM-CSF, indicating that neurodegeneration in these mice is associated with Th17 polarization (196). Increased expression of IL-17, IL-22, and RORγt has been found in the hippocampus, CSF, and serum of rats after intrathecal injection of Aβ-42 peptide (197). In the same study, Zhang et al. indicated that after disruption of the blood–brain barrier with Aβ-42 injection, Th17 cells enter into the brain (197). Tian et al. reported that postoperative cognitive dysfunction is associated with an enhanced level of IL17A in the hippocampus and suggested that IL-17-mediated damage of the hippocampus leads to Aβ1-42 accumulation and thus probably to cognitive decline (198). Increased expression of RORγt, IL-23, and IL-17 was found in the brains of Aβ-42-injected rats, while Treg-related cytokines TGF-β and IL-35 were decreased (199). Activated Th1 or Th17 cells in the brain produce inflammatory cytokines IFN-γ or IL-17 and thus heighten the inflammatory cascade, recruit and activate immune cells, and promote AD neuropathology (192, 200).

In MS, cytokines released by Th17 cells bind to their receptors on neurons and activate the apoptotic pathway, leading to neurodegeneration (201). Expression of Fas and FasL is also increased in the brain of AD rats (197, 202), and it could be assumed that Th17 cells activate the apoptotic pathway in neurons by Fas/FasL interaction and thus contribute to the development of neurodegeneration in AD (197, 203).

Elevated levels of IL-1β in the brains of AD mice homozygous for a destructive mutation of TLR4 cause up-regulation of IL-17 (204). In a very recent study, it has been shown that the administration of blocking anti-IL-17 antibody decreases the cognitive impairment and neuroinflammation induced by Aβ_1−42_ injection into cerebral ventricles of adult CD1 mice, as suggested by reduced Aβ_1−42_, glial fibrillary acidic protein (GFAP), S100 proteins, and inflammatory mediators and cytokines (205). This result supports the previously indicated role of IL-17 and related cytokines in promoting AD neuroinflammation and neurodegeneration (206). On the other hand, there is a study that indicates a protective role for IL-17 in an animal model of AD (207). Intracranially overexpressed IL-17 reduced cerebral amyloid angiopathy and improved anxiety and learning deficits (207). Further, it has recently been shown that ICR mice injected with IL-17 have an improvement in spatial learning as measured by the Morris water maze test, which is associated with the promotion of maturation of already-formed neuroblasts and the inhibition of neuroprogenitor proliferation (208).

The number of both CD4+ and CD8+ T cells in the brain parenchyma and vascular endothelium in humans with AD is higher than in healthy controls (209). Further, naive lymphocytes obtained from AD patients had increased production of Th17-related cytokine IL-21 and had higher expression of Th17 transcription factor RORγt, while monocytes obtained from the same patients produced higher amounts of IL-6 and IL-23 (210). A higher proportion of Th17 cells has been noticed in peripheral blood of patients with mild cognitive impairment due to AD pathology than in subjects with mild cognitive impairment due to pathologies other than AD and healthy controls (211). Also, higher concentrations of IL-17 and IL-23 were detected in the serum of AD patients than in healthy controls (212). IL-17 is reported to be a good plasma biomarker for distinguishing individuals with AD from cognitively healthy control subjects (213). Also, it was reported that the IL-17 concentration in cerebrospinal fluid could be used antemortem for identification of frontotemporal lobar degeneration with tau pathology (214).

It has been proposed that a desirable AD vaccine should induce Th2 and inhibit Th1/Th17 immune responses to Aβ in order to limit or prevent neuroinflammation and subsequent neurodegeneration (215).

A number of reports indicate the important role of IL-17 in AD pathogenesis; however, the precise mechanism of IL-17 upregulation in the CNS of AD patients is not known. It is possible that microbial infection, as was reported for respiratory infection (216) or inadequate immune surveillance in the gut (194), induces higher IL-17 production in the CNS, which later leads to deposition of amyloid-β. However, the opposite sequence of events is possible; that is, deposition of amyloid-β and inadequate clearance stimulate receptors of innate immune cells and induce production of IL-17, which perpetuates AD pathogenesis.

## IL-17 in Ischemic Brain Injury

Brain ischemia causes necrosis of the affected CNS tissue due to the loss of nutritional supply (217). Damaged CNS tissue releases damage-associated molecular patterns (DAMPs) that stimulate resident innate immune cells in the CNS, in the first line microglia (218). Activated microglia cells have a dual role: these cells play a beneficial role by phagocytosis of damaged tissue but also release inflammatory mediators TNF-α, IL-1β, IL-6, and IL-17, which enhance inflammation and tissue damage (219). DAMP molecules, released after ischemic brain damage, such as high mobility group 1 box 1 (HMGB1) (220, 221) and peroxiredoxin, induce IL-23 production in microglia/macrophages by activating TLR2 and TLR4, which subsequently induce the expression of IL-17 in other immune cells but also in microglia (222).

Activated immune cells after reperfusion additionally damage CNS tissue and significantly contribute to overall tissue damage after stroke. Adaptive immunity most probably contributes to inflammation development in CNS tissue after ischemia-reperfusion, especially T cells (223, 224). Similar was found in animal models: RAG1(–/–) mice, after stroke induced by transient middle cerebral artery occlusion, developed reduced damage of brain tissue, but detrimental effects of T cells in cerebral ischemia did not depend on antigen recognition or TCR costimulation (225). The presence of Th1 as well as Th17 cells was noticed in the brain lesions in ischemic stroke (226). These cells release proinflammatory cytokines and thus contribute to tissue damage (226).

Waisman et al. indicate IL-17 as one of the molecules that play a particular role in the delayed phase of the postinfarct inflammatory cascade (217). Increased expression of IL-17 mRNA in peripheral blood mononuclear cells was detected in patients after ischemic stroke, and its expression was in correlation with Scandinavian Stroke Scale scores (227). High expression of IL-17 was observed in ischemic injured brain tissue in experimental animals and also in postmortem analyzed human tissues (228–230). Also, higher expression of IL-17 at the mRNA and protein levels has been detected in the penumbral brain tissue 1, 3, and 6 days after reperfusion in mice (231). In an animal model of ischemic stroke, an increased number of IL-17-producing blood mononuclear cells were observed (232). In another study, IL-17 levels were elevated 3 days after reperfusion. This induction of IL-17 production was IL-23-dependent, and γδ T cells were indicated as the main source of IL-17 (233). In this study, it has been shown that IL-17 plays the main role in the stage of tissue damage after infarction, since IL-17-deficient mice had attenuated damage only on day 4 after ischemic insult (234). On the other hand, IL-23p19-deficient mice developed attenuated CNS tissue damage on day one after stroke induction (234), but γδ T-cell-deficient mice still develop brain injury after ischemia reperfusion induction (228). Although there is no clear evidence that Th17 cells play a role in tissue damage after stroke induction, activation of T cells and autoantigen-specific T cells, which exacerbates ischemic brain injury, was noticed in experimental animals (234). Also, an increase in the proportion of Th17 cells and a decrease in Treg cells in the periphery might contribute to CNS tissue damage after ischemia-reperfusion (235). Astrocytes are also marked as a source of IL-17 in inflammatory foci after brain ischemia-reperfusion (228, 236).

IL-17 may contribute to CNS tissue damage by several mechanisms, as described previously in other inflammatory diseases of CNS, affecting the cells that express IL-17 receptor, microglia, endothelial cells, astrocytes, and neurons, as summarized in Table 1. Although ischemia-reperfusion induces necrosis of blood–brain barrier, IL-17 could enhance BBB damage by the disruption of tight junctions (120) and by the promotion of monocyte migration across the BBB through an intracellular adhesion molecule (ICAM) 1-dependent mechanism (159). Levels of inflammatory cytokines IL-1β, TNFα, and matrix metalloproteinases, indicators of BBB damage, are decreased after stroke induction in IL-17-deficient mice (233). Increased expression of IL-17 receptor on neurons has been shown simultaneously with increased expression of IL-17 in CNS tissue after stroke induction, indicating the role of IL-17 in direct neuronal damage (230). This observation is supported by *in vitro* study (230). IL-17 also enhances autophagy in neurons and thus aggravates neuronal ischemic injuries (237). In synergy with TNF-α released by macrophages, IL-17 stimulates astrocytes to produce CXCL1, which recruits neutrophils into the CNS and thus enhances inflammation and damage (228). Astrocytes stimulated *in vitro* with TNFα and IL-17A show enhanced expression of several chemokines that have a role in the attraction of other immune cells, CCL20, CXCL2, CXCL9, CXCL10, and CXCL11, (153). IL-17, synergistically with IL-6, induces expression of CCL20 in astrocytes, which is a chemokine that attracts Th17 cells (149).

**Table 1:** The main cellular source of IL-17 and its target cells in chronic inflammatory neurological diseases.

	**Multiple sclerosis**	**Alzheimer's disease**	**Ischemic brain injury**
Main source of IL-17	Th17	Neutrophils	γδ T cells
IL-17 target cells	• Astrocytes • BBB endothelial cells • Microglia/macrophages • Oligodendrocyte precursor cells	• Neutrophils • Neurons • Microglia/macrophages • BBB endothelial cells	• BBB endothelial cells • Astrocytes • Neurons • Microglia/macrophages
Main biological effects of IL-17	• BBB disruption • Induction of inflammation • Myelin damage	• Induction of inflammation • Deposition of amyloid-β	• BBB disruption • Induction of inflammation • CNS tissue damage

*BBB, Blood–Brain Barrier*.

On the other hand, IL-17A induces the expression of molecules that have neuroprotective effects, brain-derived neurotrophic factors (BDNF), glia-derived neurotrophic factors (GDNF), and nerve growth factors (NGF), indicating that IL-17 might have a role in the reduction of damage (157). Recently, it has been shown that recombinant mouse IL-17A significantly attenuates damage of cortical astrocytes after stroke induction in a dose-dependent manner by inhibition of apoptosis (238).

## Concluding Comments

**Figure 1:**
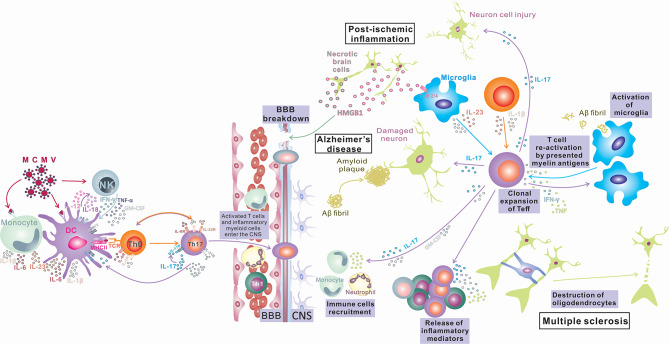
IL-17 in Inflammatory Diseases of the Central Nervous System. Infection in the periphery (viral infections) activates innate immunity (monocytes/macrophages and NK cells) and induces a proinflammatory environment that changes the phenotype of antigen-presenting cells, which differentiate into inflammatory APCs that produce inflammatory cytokines IL-1, IL-6, IL-12, and IL-23. These APCs induce bystander activation of autoreactive T cells and their differentiation toward encephalitogenic T cells (IFN-γ, IL-17, TNF-α, Tbet, RORγt, CXCR3, and CCR6 positive) capable of entering the CNS, where after reactivation in contact with antigens presented by local tissue APCs, they proliferate and produce cytokines (IL-17, GM-CSF) that contribute to BBB disruption and recruitment of other immune cells into the CNS, finally inducing myelin damage (Multiple sclerosis). Peripheral infections can compromise the BBB and lead to an influx of IL-17-producing cells into the CNS. IL-17 can induce damage to neurons by direct cytotoxic effects or by recruitment of neutrophils and induction of inflammation, leading to deposition of amyloid fibrils and plaque formation (Alzheimer's disease). Also, the opposite order of events is possible, where microglia phagocytize amyloid fibrils and induce differentiation of T cells toward IL-17-producing cells, and the released IL-17 damages the BBB, recruits neutrophils, and induces inflammation and neuron damage, which exacerbates amyloid deposition (Alzheimer's disease). CNS tissue damaged by ischemia releases damage-associated molecular patterns (HMGB1) that stimulate microglia to release inflammatory mediators TNF-α, IL-1β, IL-6, and IL-17, which enhance inflammation and tissue damage. Activated microglia also can induce Th17 development. IL-17 released by innate immune cells or Th17 cells can enhance BBB damage, recruit immune cells, and enhance inflammation, inducing direct neuronal damage (postischemic inflammation).

Despite a large number of reports that indicate an important or, in some diseases, even indispensable role of IL-17 and Th17-related cytokines in inflammatory and degenerative neurological diseases, the precise mechanism of the pathogenic effect of IL-17 in the CNS is still elusive. Numerous *in vivo* and *in vitro* studies identify several types of CNS tissue cells as IL-17 targets and illustrate the effects of stimulation of these cells with IL-17 (summarized in Figure 1). However, the relative contributions of these processes to tissue damage and the development of inflammatory CNS diseases in humans are still undetermined. In order to gain new insights into the role of IL-17 in the pathogenesis and eventual new treatment of neuroinflammatory and neurodegenerative diseases, additional research in this field is required.

## Author Contributions

All authors listed have made a substantial, direct and intellectual contribution to the work, and approved it for publication.

## Conflict of Interest

The authors declare that the research was conducted in the absence of any commercial or financial relationships that could be construed as a potential conflict of interest.
